# Prevention and care of paediatric HIV infection in Ouagadougou, Burkina Faso: knowledge, attitudes and practices of the caregivers

**DOI:** 10.1186/s12887-016-0569-y

**Published:** 2016-03-09

**Authors:** Malik Coulibaly, Elisabeth Thio, Caroline Yonaba, Sylvie Ouédraogo, Nicolas Meda, Fla Kouéta, Désiré Lucien Dahourou, Angèle Kalmogho, Mady Gansonré, Diarra Yé, Ludovic Kam, Valériane Leroy, for the MONOD Study Group*

**Affiliations:** Projet MONOD, ANRS 12206, Centre de Recherche Internationale pour la Santé, 09 BP 168 Ouagadougou, Burkina Faso; Centre Muraz, Bobo Dioulasso, Burkina Faso; CHU Charles De Gaules, Service de Pédiatrie médicale, Ouagadougou, Burkina Faso; CHU Yalgado Ouédraogo, Service de Pédiatrie, Ouagadougou, Burkina Faso; Inserm U1219, Institut de Santé Publique, Epidémiologie et Développement, Université de Bordeaux, Bordeaux, France; Inserm U1027 Université Paul Sabatier, Toulouse 3, Toulouse, France

**Keywords:** HIV infection, Caregivers, Early infant diagnosis, Paediatrics, Africa

## Abstract

**Background:**

The paediatric Human Immunodeficiency Virus (HIV) epidemic still progresses because of operational challenges in implementing prevention of mother-to-child HIV transmission (PMCT) programs. We assessed the knowledge, attitudes and practices (KAP) of children’s caregivers regarding mother-to-child transmission (MTCT) of HIV, paediatric HIV infection, early infant diagnosis (EID), and paediatric antiretroviral treatment in Ouagadougou, Burkina Faso.

**Methods:**

We undertook a qualitative survey in the four public hospitals managing HIV exposed or infected children, in Ouagadougou in 2011. A sociologist used a semi-structured questionnaire to interview caregivers of children less than 5 years old attending the paediatrics wards on their KAP. Study participants were divided into four groups as follows:

those who did not yet know their children’s HIV infection status, those who were waiting for their children’s HIV test results, those who were waiting for antiretroviral treatment, and those who were already on antiretroviral treatment.

**Results:**

A total of 37 caregivers were interviewed. The mean age was 32.5 years, and 29 (78 %) were mothers. Twenty seven (73 %) caregivers had primary or higher level of education, and 15 (40 %) described their occupation as “housewife”. Overall, 36 (97 %) of caregivers knew that the main route of HIV transmission for infants was through MTCT and 14 (38 %) specified that it occurred during pregnancy or delivery. Five percent thought that MTCT of HIV occurred during conception. PMTCT interventions could help prevent infant HIV infection according to 32 (87 %) caregivers. Thirty five percent of caregivers stated EID as a prevention strategy. Fifty-four percent of the participants believed that replacement feeding option would prevent MTCT of HIV; 24 (65 %) stated that they would prefer medical practitioners seek caregivers’ consent before carrying out any HIV-test for their child, and that caregivers’ consent was not compulsory before antiretroviral treatment. All caregivers thought that it was necessary to treat HIV-infected children, although they did not know what interventions could be done.

****Conclusion**s:**

This study highlighted the low level of caregivers’ knowledge on paediatric HIV prevention and care in Ouagadougou. Awareness programs targeting caregivers need to be strengthened in order to improve the uptake of HIV early infant diagnosis and care.

**Electronic supplementary material:**

The online version of this article (doi:10.1186/s12887-016-0569-y) contains supplementary material, which is available to authorized users.

## Background

An estimated 3.3 million of children under 15 years of age are currently living with Human Immunodeficiency Virus (HIV) worldwide, with the majority in sub-Saharan Africa [1]. The infant mortality among perinatally HIV-infected children is high and can reach 52 % at 2 years of age in the absence of antiretroviral treatment [2, 3]. In 2008, the World Health Organization (WHO) recommended the universal early antiretroviral treatment (ART) for all HIV-infected children less than 12 months of age, irrespective of clinical status or symptomatology, because early infant antiretroviral treatment has proved to be efficient in improving their survival [4]. This recommendation was extended to all children less than 24 months of age in 2010 [5]. However, only 34 % of eligible children for treatment were actually on ART at the end of 2012, compared with a 63 % adult coverage, and only 35 % of infants born to HIV-positive mothers had an early infant HIV diagnosis (EID) in their first 2 months of life [1]. This inequity in access to EID and ART in children is worst in West Africa, compared to East and Southern Africa [6].

To improve early access to paediatric antiretroviral therapy in resource-limited settings, it is crucial to implement public health strategies to promote the acceptability of HIV EID at the community level, targeting both health care professionals and children’s caregivers [7]. This is even truer in West Africa [8, 9].

Thus, it is crucial to understand caregivers’ knowledge and perception in order to understand the barriers to paediatric HIV prevention and treatment strategies. Caregivers’ knowledge regarding paediatric HIV care is important for children’s antiretroviral treatment adherence as reported elsewhere [10–14].

Burkina Faso is a West African developing country with a gross national income per capita of $1,500 in 2013 [15]. The overall schooling rate at primary school in 2009/2010 was estimated at 74.8 % [16]. The public health system was divided into three levels: the health districts, the regional hospitals, and the university hospitals. In addition, there were 398 private health care facilities operating in the country in 2012 [17]. The 2010 health and demographic survey reported a national HIV prevalence of 1.0 %, reaching 2.1 % (sample size 2018) in Ouagadougou [18].

The 2010 health demographic survey also revealed that 98.3 % of women (sample size 17, 087) and 98.1 % of men, aged from 15 to 49 years (sample size 6,500) were aware of Acquired Immunodeficiency Syndrome (AIDS) in the whole country. In Ouagadougou, the capital of the country, 99.4 % (sample size 2,240) of women and 99.8 % (sample size 977) men were aware of AIDS. The use of condom as a mean of HIV prevention was known in 78.0 % (sample size 17,087) of women and 90.1 % (sample size 6,500) of men, aged from 15 to 49 years in the country. Knowledge related to the limited sexual intercourses to a single HIV-uninfected partner as a mean of prevention of sexual transmission, was mentioned by 86.1 % of women and 91.6 % of men aged from 15 to 49 years [18]. According to a study conducted in 2009 in Bobo Dioulasso, only 27.3 % (82/300) of women were aware of HIV MTCT through breastfeeding [19].

In Ouagadougou, Burkina Faso, it has been reported that 71 % of HIV exposed children did not have access to EID in 2011 [20]. Several factors are associated with this big gap: poor health facilities, health workers’ and caregivers’ low level of knowledge, negative attitudes and bad practices. In 2014, few data were available on caregivers’ knowledge about EID and paediatric HIV care in Burkina Faso’s field conditions. Thus, the objective of our study was to assess the knowledge, attitudes, and practices of the parents/caregivers regarding mother-to-child-transmission (MTCT) of HIV infection, EID, paediatric HIV infection, and antiretroviral treatment for HIV-infected children in Ouagadougou in 2011.

## Methods

### Study setting

The present study was carried out in the only four public hospitals providing antiretroviral treatment to HIV-infected children in Ouagadougou. Two of them were university hospitals: Yalgado Ouédraogo (YO) university hospital and Charles De Gaulle (CDG) paediatric university hospital. YO was the country’s largest referral hospital and CDG the national referral paediatric hospital.

The two other study sites were district hospitals (Boulmiougou and Nongr-Massom) which also served as reference hospitals for their respective health districts. Some of the study sites characteristics are described in the Table 1.

**Table 1:** Characteristics of study site hospitals in Ouagadougou, Burkina Faso, 2011 [26]

	Physicians	Pharmacists	Nurses	Mid-wives	Beds in paediatric wards	Total beds in wards	Bed occupancy rate (%)
Yalgado Ouédraogo university hospital	128	13	113	35	112	733	88.9
Chrales De Gaulle peadiatric university hospital	23	3	57	0	143	143	61.2
Boulmiougou district hospital	10	3	80	41	35	62	52.3
Nongr-Massom district hospital	13	1	40	26	24	95	0.7

Bed occupancy rate = number of days of inpatient care/number of bed days available

Number of days of inpatient care = total number of days in which each patient occupied a bed in a health facility over the year

Number of bed days available = number of available beds in the health facility X the number of days in the year

Our study was part of a larger multidisciplinary (sociology, immunology, virology, pharmacokinetics, nutrition) research project aimed to improve EID and ART initiation with lopinavir/ritonavir before the age of 2 years among HIV-infected children, and to assess strategies for the simplification of ARV among children who had achieved virological suppression after 1 year of ART (the MONOD ANRS 12206 project).

### Study design

We conducted a 2-week qualitative cross-sectional survey in January 2011 in Ouagadougou. Based on the 2010’s Ministry of health records, we selected the four public hospitals which provided paediatric HIV care in Ouagadougou in 2010 [20]. In order to represent all the stages of paediatric HIV cascade of care, we chose to have four groups of caregivers in our sample: caregivers’ children HIV status unknown, caregivers’ children HIV test result pending, caregivers’ children HIV infection confirmed but untreated with antiretroviral drugs, and caregivers’ HIV-infected children treated with ART. In the first place, caregivers were identified in the paediatric wards of the study sites, especially in the units where the HIV-infected children were treated. Then, the sociologist visited the symptomatic children (severe malnutrition for instance) seeking the children already screened for HIV but who were not yet on antiretroviral treatment. Lastly, she looked among the outpatient caregivers consulting in paediatric wards, for those whose children’s HIV infection status was unknown. This group of “unknown HIV infection status” represented the general population of children’s caregivers. In each unit visited, all caregivers with children less than 5 years old were contacted for enrolment in the study. Those who consented were face-to-face interviewed using a semi-structured questionnaire. To maintain confidentiality, each caregiver was interviewed in a room. The sociologist who carried out the interview had been trained and had experience in HIV/AIDS care. The interviews were done in French and in Mooré (a national language), languages in which the sociologist was fluent.

The answers were tape-recorded, further transcribed, and analysed both manually by the sociologist, and with Stata software for the quantitative data. The comparison between groups was carried out using Pearson chi^2^ or Fisher’s exact test if at least one expected frequency was < 5. The confidence interval was set at 95 % and the significance *P*-value < 0.05.

The questionnaire was designed to collect the caregivers’ knowledge, attitudes and practices about prevention of HIV infection, MTCT, EID, paediatric HIV infection, and antiretroviral treatment for HIV-infected children in Ouagadougou.

The study was implemented as a situational analysis of the ANRS 12206 MONOD trial (ClinicalTrial.gov registry n°NCT01127204) and approved by the Ethics committee for health research and the Health Ministry of Burkina Faso. All the interviewees gave their verbal clear consent.

## Results

Overall, 47 caregivers of eligible children were enrolled in the study, of whom 37 (79 %) consented to be interviewed. The refusal proportion was 21 % (10/47). Among the caregivers who accepted the interview, the mean age was 32.5 years ± 7 years, 29 (78 %) were women, and 31 (84 %) lived with their partner. Among these 29 women, 28 were the mothers and the remaining one was an aunt. Fathers were 8 (22 %). Twenty-seven (73 %) had primary or higher education, and 15 (40 %) described their occupation as “housewife” (Table 2). Each caregiver interviewed belonged to one of the four groups of HIV paediatric care: caregivers whose HIV-infected child was on ART (*n* = 11), caregivers whose child was known to be HIV-infected but not yet on ART (*n* = 4), caregivers waiting for their child’s HIV test results (*n* = 5), and those just attending outpatient paediatric ward with an unknown HIV status child (*n* = 17), as described in Table 2.

**Table 2:** Socio-demographic description of children’s caregivers according to their child HIV status, Ouagadougou, Burkina Faso, 2011

	Total *N* = 37 (%) 100 %	Group 1 *N* = 11 (%) 100 %	Group 2 *N* = 4 (%) 100 %	Group 3 *N* = 5 (%) 100 %	Group 4 *N* = 17 (%) 100 %
Mean age (years)	32.5 ± 1.1	31.5 ± 2.0	35 ± 4.0	29.6 ± 4.4	33.4 ± 1.6
Education					
No schooling	10 (27)	5 (46)	1 (25)	3 (60)	1 (6)
Primary	7 (19)	3 (27)	1 (25)	2 (40)	1 (6)
Secondary	11 (30)	2 (18)	2 (50)	0 (0)	7 (41)
Post-secondary	9 (24)	1 (9)	0 (0)	0 (0)	8 (47)
Occupation					
House wives	15 (40)	6 (55)	1 (25)	3 (60)	5 (29)
Students	3 (9)	0 (0)	0 (0)	0 (0)	3 (18)
Civil servant	4 (11)	0 (0)	0 (0)	0 (0)	4 (24)
Private sector	15 (40)	5 (45)	3 (75)	2 (40)	5 (29)
Sex					
Male	8 (22)	0 (0)	1 (25)	1 (20)	6 (35)
Female	29 (78)	11 (100)	3 (75)	4 (80)	11 (65)
Marital status					
Single	1 (3)	0 (0)	0 (0)	0 (0)	1 (6)
Married/co-habiting	31 (84)	10 (90)	2 (50)	4 (80)	15 (88)
Widowed/separated	5 (13)	1 (10)	2 (50)	1 (20)	1 (6)

Group 1: caregivers of HIV-infected child currently treated with antiretroviral therapy

Group 2: caregivers of HIV-infected child not yet initiated on antiretroviral therapy

Group 3: caregivers waiting for their child’s HIV post-test result

Group 4: caregivers attending paediatric ward, with an unknown HIV child status

### Knowledge on prevention of mother to-child-transmission (PMTCT), EID, and treatment of HIV

Overall, 97 % of caregivers knew that the main transmission route for HIV-infected infants was through their mothers. Fourteen (38 %) specified that mother-to-child transmission occurred during pregnancy or delivery. For instance a mother stated: “*if the mother ignores her own HIV infection status, or if she is aware of it and does not benefit from mother-to-child HIV prevention treatment, she can infect her foetus during pregnancy*”. Another caregiver said: “*during delivery if the delivery table and the materials used on an HIV-infected woman is not well cleaned*”. Some of them declared: “*during delivery if there are wounds*” or “*a child can be infected when the umbilical cord is cut*”. Five percent of caregivers thought that infant HIV transmission can occur during sexual intercourses, at the time of conception. For instance, a caregiver said that “*an infant can get the disease through sexual intercourse (at conception) if the parents are HIV-infected*”.

To the question about how can we prevent children’s HIV infection, 87 % of the caregivers responded that testing mothers and providing antiretroviral drugs to the HIV-infected ones (PMTCT interventions) can help prevent children’s HIV infection. A caregiver mentioned: “*to protect infants from the disease, when a woman is pregnant, she must attend a health centre for HIV testing, and if she is infected, she should be given some drugs to prevent her infant from being infected*”. Twenty (54 %) were aware that breast milk substitutes could prevent HIV transmission.

For EID, 32 (86 %) had a general knowledge on diagnostic method using blood sample, among them those attending paediatric ward with an unknown HIV status child were the most represented (Table 2). However, 6 (16 %) of caregivers felt that the EID was not necessary if the child was not symptomatic; 24 (65 %) stated that health workers should obtain caregivers’ consent before carrying out any HIV-test. *“Concerning infant HIV diagnostic, health worker should not seek caregivers’ consent, but they can inform them*” said another caregiver. Infant HIV diagnosis should be motivated by the child’s recurrent illnesses, for 10 (27 %) caregivers, of whom 4 were fathers and 6 mothers. “*I have not performed HIV test for my child yet, because he is healthy, he is not frequently sick*”, said one parent.

As for antiretroviral treatment, 24 (65 %) thought that caregivers’ consent is not compulsory before treating an HIV-infected child with antiretroviral drugs. “A caregiver stated that “*doctors must not seek caregivers’ consent, because they are the ones who know what is good for the child*”. The caregivers’ knowledge perception of consent is summarised in Table 3.

**Table 3:** Caregivers’ knowledge, attitudes and perceptions in Ouagadougou, Burkina Faso, 2011

	Total *N* = 37 100 %	Group 1 *N* = 11 100 %	Group 2 *N* = 4 100 %	Group 3 *N* = 5 100 %	Group 4 *N* = 17 100 %	Groups 1 + 2 + 3 *N* = 20 100 %	*P*-value (Group 1 + 2 + 3 vs Group 4)
Caregiver’s knowledge of existing interventions to prevent MTCT of HIV							
Yes	30 (81)	7 (64)	4 (100)	3 (60)	16 (94)	14 (70)	0.16
No	1 (3)	0 (0)	0 (0)	1 (20)	0 (0)	1 (5)	
No response	6 (16)	4 (36)	0 (0)	1 (20)	1 (6)	5 (25)	
Caregiver’s knowledge regarding existing methods of infant HIV diagnosis							
Yes	32 (86)	8 (73)	4 (100)	5 (100)	15 (88)	17 (85)	0.77
No	5 (14)	3 (27)	0 (0)	0 (0)	2 (12)	3 (15)	
Caregiver’s knowledge regarding existing treatment of HIV-infected infants							
Yes	37 (100)	11 (100)	4 (100)	5 (100)	17 (100)		Not applicable
No	0 (0)	0 (0)	0 (0)	0 (0)	0 (0)		
Caregiver’s attitude regarding the practice of their child systematic HIV testing							
For	31 (84)	11 (100)	3 (75)	4 (80)	13 (76)	18 (90)	0.26
Against	6 (16)	0 (0)	1 (25)	1 (20)	4 (24)	2 (10)	
Caregiver’s attitude regarding the antiretroviral treatment of HIV-infected children							
For	37 (100)	11 (100)	4 (100)	5 (100)	17 (100)	20 (100)	Not applicable
Against	0 (0)	0 (0)	0 (0)	0 (0.0)	0 (0.0)	0 (0)	
Parent’s consent needed for child HIV-test							
Yes	24 (65)	8 (73)	3 (75)	4 (80)	9 (53)	15 (75)	0.16
No	13 (35)	3 (27)	1 (25)	1 (20)	8 (47)	5 (25)	
Parent’s consent needed for child treatment							
Yes	11 (30)	6 (55)	0 (0)	1 (20)	4 (24)	7 (35)	0.25
No	24 (65)	4 (36)	3 (75)	4 (80)	13 (76)	11 (55)	
No response	2 (5)	1 (9)	1 (25)	0 (0)	0 (0)	2 (10)	

Group 1: caregivers of HIV-infected child currently treated with antiretroviral therapy

Group 2: caregivers of HIV-infected child not yet initiated on antiretroviral therapy

Group 3: caregivers waiting for their child’s HIV post-test result

Group 4: caregivers attending paediatric ward, with an unknown HIV child status

*Vs* versus

Finally, for HIV infection management, all caregivers thought that it was necessary to treat HIV-infected children, although they did not know what type of interventions should be done. A caregiver said: “*it is important to treat HIV-infected children because it makes them healthier. See, my daughter has regained health and she is playing*”.

PMTCT interventions included EID for only 13 (35 %) caregivers.

There was no statistical significant difference between groups of caregiver’s whose children were already HIV tested (groups 1 + 2 + 3) and those coming for outpatient consultation (group 4) regarding knowledge and attitudes (Table 3).

### Practice of children’s HIV testing

Among the twenty participants (51.1 %) who carried out the HIV test of their children, 17 (45.9 %) revealed that the major reason underlying their decision was the frequent illnesses of their children. For those who tested their children because of illness symptoms, two mentioned additional reasons for testing: PMTCT intervention and the death of the child’s mother. Furthermore two (5.4 %) caregivers admitted that they tested their children because of the positive HIV status of the parents. One (2.7 %) caregiver did not specify his motivation for carrying out the HIV test.

One mother revealed that her child was tested without her consent.

Among the 17 (48.9 %) caregivers who had not tested their children, six (16.6 %) justified their attitude by the mother’s negative test result after the prenatal HIV testing. The other reasons for not testing the children were: healthy appearance (2.7 %) of the child and unawareness of the possibility of carrying out the test of the child without a medical prescription (2.7 %). In addition, one (2.7 %) caregiver said: “*my child is three years old, he is still young and he can do his HIV test later*”. The eight (21.6 %) remaining caregivers who had not tested their children provided no explanation.

## Discussion

This survey gave an insight of caregivers’ knowledge and attitude about PMTCT, paediatric HIV infection diagnostic, existing prevention and HIV treatment strategies for children in Ouagadougou in 2011. Firstly, this study showed that despite an overall knowledge of MTCT modes, a few misconceptions still existed. Secondly, we highlighted the low level of knowledge among caregivers about the importance of performing EID even in asymptomatic HIV-exposed children allowing an earlier access to ART. Our results are useful to understand the barriers of EID uptake and paediatric HIV care in West-African children.

A total of 21 % of caregivers refused to participate in this survey, highlighting the possible lack of confidentiality and fear of stigma already mentioned in a similar context [21]. This lack of confidentiality may have also influenced the information collected among the responders, mainly in minimizing their difficulties facing the health care system or the health care workers.

Nevertheless, we were able to understand to some extent, caregivers’ knowledge, attitudes and practices at the different stages of paediatric HIV care cascade.

Our results showed that caregivers were globally aware of the HIV infection disease. We assume that this is the result of HIV prevention campaigns at community level in Burkina Faso since the early years when first cases of HIV infection were reported [22, 23].

Similarly, knowledge about PMTCT and EID using blood samples method was appreciable among caregivers, and there was no statistically significant difference between the groups when it comes to the method used to test their children. This result could be related either to a lack of statistical power, or to an absence of difference in terms of knowledge attitude and practices. However, as we expected a difference in knowledge about PMTCT and EID method in favour of those who were already in contact with HIV health care providers (groups 1 + 2 + 3), because of their frequent interactions with health staff, we could raise the hypothesis that health visits were not efficiently utilized to raise their awareness. So, health care providers should take advantage of the medical visits to cope with these issues.

But, the lack of difference in terms of knowledge of the group 4 versus the groups 1 + 2 + 3 can also be related to the level of education: the level of education amongst group 4 is greatly elevated and could account for any of the differences seen between the two groups.

In addition, caregivers misunderstanding and low level of knowledge about EID in asymptomatic children still remained. This could impede the routine EID coverage, and ultimately delay the ART treatment. This could partially explain the EID bottleneck in the 2011 cascade of care in Ouagadougou, with only 29 % of HIV-exposed children testing within their fist year of life [21]. Improving the coverage of the paediatric HIV care cascade at each stage requires addressing these misunderstandings through awareness campaigns. For instance in South Africa, a study showed that EID rate can be improved by using some strategies including awareness campaigns [24].

Some caregivers were reluctant to the practice of an EID without their consent, highlighting the fear of a possible stigmatization or mistrust of health workers’ practices. Conversely, they did not perceive the importance of consent prior to the infant antiretroviral treatment, whereas this formal consent would increase caregivers’ adherence to their infant ARV treatment [11, 14].

It is interesting to notice that all of the caregivers thought that early antiretroviral treatment was necessary for all HIV-infected infants, and this would decrease the mortality and morbidity rate if the service was accessible to the population. This could explain why we recorded a small proportion of refusal among caregivers referred for their infant HIV treatment initiation in the recruitment phase of the MONOD trial, implemented in Ouagadougou. The results of this trial will be presented elsewhere.

Some issues related to staff practices, such as carrying out the HIV test without caregivers’ prior consent, may be addressed by improving training in paediatric wards, as it has already been reported in Côte d’Ivoire [9] and South Africa [25]. However, changing caregivers’ attitudes may require interventions to raise their awareness at both individual and community level.

As for healthcare providers, they should actively promote HIV testing and care-seeking for children, by seizing all opportunities with caregivers.

Before concluding, we could discuss a few study limits. Although, our sample size was limited, the sampling method was adapted to qualitatively understand some perceptions, at the different steps of the cascade of paediatric HIV care from access to PMTCT services, EID services, and child care in Ouagadougou.

In addition, considering a group of “caregivers of HIV exposed but confirmed uninfected children” could have enriched the understanding of KAP in Ouagadougou, but it was not feasible in the cross-sectional design at the time of attendance in paediatric care.

## Conclusions

In conclusion, although awareness of paediatric HIV infection among caregivers is significant in Ouagadougou, some misconceptions related to the moment of transmission and the importance of performing routine EID in all HIV-exposed children, still persist. These issues must be addressed by strengthening awareness campaigns and promoting best practices among medical staff. Therefore, changing caregivers’ attitudes may require interventions at both individual and community level. Healthcare providers should actively promote HIV testing and care-seeking for children in order to improve the uptake of EID among HIV-exposed children in Burkina Faso.

## Additional file

Additional file 1: Definition of the ANRS 12206 MONOD Collaboration Study Group. (DOC 31 kb)

## Abbreviations

AIDS: acquired immunodeficiency syndrome
ANRS: Agence Nationale de Recherche pour le SIDA et les hépatites virales (French National Agency for AIDS and viral hepatitis)
ART: antiretroviral treatment
CRP-Santé: centre de recherche publique pour la Santé (Public Research Centre for Health)
EDCTP: European and developing countries clinical trial partnership
EID: early infant diagnosis
HIV: human immunodeficiency virus
KAP: knowledge, attitudes and practices
MTCT: mother-to-child transmission
PMTCT: prevention of mother-to-child transmission
Vs: versus
WHO: world health organisation
