# Supplementary material for: Prevention and care of paediatric HIV infection in Ouagadougou, Burkina Faso: knowledge, attitudes and practices of the caregivers
Source: BMC Pediatr. 2016 Mar 9;16:33. doi: 10.1186/s12887-016-0569-y (PMC4784410; doi:10.1186/s12887-016-0569-y)
Supplement: Additional file 1: — Definition of the ANRS 12206 MONOD Collaboration Study Group. (DOC 31 kb) [file 12887_2016_569_MOESM1_ESM.doc]

**Additional file**

**Additional file 1:** Definition of the ANRS 12206 MONOD Collaboration Study Group

**Participating sites**

**Burkina Faso, Ouagadougou:** Centre de Recherche Internationale pour la Santé: Malik Coulibaly, Désiré Lucien Dahourou, Nicolas Meda (**co-investigator**) Colette Ouédraogo, Mamadou Sawadogo, Wilfried Somé, Désiré Sondo, Elisabeth Thio. CHU Charles De Gaulle : Mamoudou Barry, William Hiembo, Fla Kouéta, Adama Ouattara, Moussa Ouédraogo, Rasmata Ouédraogo, Sylvie Ouédraogo, Bernadette Congo, Rose Barry, Diarra Yé, CHU Yalgado Ouédraogo: Malika Congo, Edouard Minéné, Marie Coulibaly, Pierre Innocent Guissou Angèle Kalmogho, Ludovic Kam, Emile Ouédraogo, Lassana Sangaré, Caroline Yonaba. Programme Sectoriel Santé de Lutte contre le SIDA et les IST : Ramatou Sawadogo. Programme d’Appui au Monde Associatif et Communautaire (PAMAC) : Odette Ky-Zerbo.

**Côte d’Ivoire, Abidjan:** Programme PACCI: Xavier Anglaret, Clarisse Amani-Bossé, Divine Avit, Christine Danel, Serge Eholié, Didier Ekouévi, Eulalie Kanga, Suzanne Kouadio, Séverin Lennaud, Maxime Aimé Oga, Thérèse N’Dri-Yoman. CHU Cocody: Madeleine Amorissani-Folquet, Evelyne Dainguy, Beugre Kouassi, Jean-Claude Kouassi, Gladys Oka. CHU Yopougon : Kader Keita, Jean Yves Lambin, François Eboua Tanoh, Marguerite Timité-Konan (**co-investigator**). Site Abobo-Avocatier : Véronique Mea-Assande, Site CePReF-enfants : Addi Edmond Aka, Hortense Aka-Dago, Sylvie N’Gbeche, Eugène Messou. Lab CeDReS : Arlette Emieme, Fatoumata Koné, Hervé Menan, Thomas Toni, Vincent Yapo. Programme National de Prise en Charge: Kouamé Abo, Irma Ahoba, David Aka. FSU Abobo-Avocatier: Gbaméné Kouassi. Pharmacie de la Santé Publique : Carine Kodo ; Implementers : Touré Siaka, Pety Touré (ACONDA), Fassinou Ekouevi (EGPAF), Ida Viho (ICAP), Anthony Richard Tanoh, Olivier Blé (Fondation ARIEL GLASER). Community representants: Yaya Coulibaly (RIP+), Philomène Takouo (ONG Bayema). Programme ESTHER: Jean Marie Massumbuko. CIRBA: Kouadio Kouakou, Programme National de Santé Infantile: Dorothée Koumi, Programme Elargi de Vaccination: Berté Koné.

**Methodology and Data Management Center : Inserm U897, Institut de Santé Publique, d'Épidémiologie et de Développement, University of Bordeaux, France**: Sophie Dattez, Sophie Karcher, Jérôme Le Carrou, Valériane Leroy (**Coordinating investigator**), Karen Malateste, Pierre Touret. Methodological Support: Caroline Bouyssou, Geneviève Chêne, Valérie Conte, Sophie Desmonde, Delphine Gabillard, Valérie Journot, Roger Salamon. MEREVA, Bordeaux. **Website:** <http://mereva.isped.u-bordeaux2.fr/monod/Accueil.aspx>

**Supporting teams:**

CRP-Santé, Luxembourg: Vic Arendt (**co-investigator**), Carole Devaux, Jean-Claude Schmidt.

CHU HUDERF, Bruxelles, Belgique: Philippe Lepage (**co-investigator**)

EA 3620, Université Paris Descartes, Paris, France: Stéphane Blanche (**co-investigator**), Marie-Laure Chaix-Baudier, Deborah Hirt, Christine Rouzioux, Alain Pruvost (CEA), Jean-Marc Treluyer, Saik Urien.

Inserm U1058, Université Montpellier 1, France: Philippe Van de Perre (**co-investigator**).

**Administrative Team:** Elodie Vernoux (Bordeaux, France), Aminata Paré-Karambiri (Ouagadougou, Burkina Faso), Zouma Tinto (Ouagadougou, Burkina Faso), Adoulaye CISSE (Abidjan, Côte d’Ivoire), Madikona Dosso (Abidjan, Côte d’Ivoire)

**MONOD ANRS 12206 Scientific Steering Committee:** Roger Salamon (Chair, Bordeaux, France), Valériane Leroy (Coordinating investigator, Bordeaux, France), Nicolas Meda (Co-Investigator, Ouagadougou, Burkina Faso), Marguerite Timite-Konan (Co-Investigator, Abidjan, Côte d’Ivoire), Vic Arendt (Co-Investigateur, Luxembourg), Stéphane Blanche (Co-Investigator, Paris, France), Philippe Lepage (Co-Investigator, Bruxelles, Belgique), Philippe Van de Perre (Co-Investigator, Montpellier, France), François Dabis (Bordeaux, France), Jean-Claude Schmidt (CRP-Santé, Luxembourg).

**MONOD ANRS 12206 trial independent data monitoring committee meeting:** Dominique Costagliola (Chair, Paris, France), Mark Cotton (Cape Town, South Africa), Carlo Giaquito (Bologna, Italie), Diana Gibb (London, UK), Elisabeth Menu (Paris, France).

**Promotor:** Inserm-ANRS, France: Jean-François Delfraissy (Director), Brigitte Bazin, Marie de Solère, Claire Rekacewicz.

**Funding:** Centre pour la Recherche en Santé (CRP-Santé), Luxembourg, European and Developing Countries Clinical Trials Partnership (EDCTP, reference: IP.2007.33011.002), French INSERM-ANRS, Institut de Santé Publique, d'Épidémiologie et de Développement, University of Bordeaux, France.

**ClinicalTrial.gov registry n°NCT01127204.**

The content is solely the responsibility of the authors and does not necessarily represent the official views of the French INSERM-ANRS, EDCTP, or University of Bordeaux.
